# Effect of nanoencapsulation on the phenomenon of drug interaction between anti-*M.avium* drug, rifabutin and anti-HIV drug, ritonavir by employing poly (lactide-co-glycolide) nanoparticles

**DOI:** 10.1186/1471-2334-12-S1-P13

**Published:** 2012-05-04

**Authors:** Tapinder Grewal, Sadhna Sharma

**Affiliations:** 1 Department of Biochemistry, Postgraduate Institute of Medical Education and Research, Chandigarh, India

## Background

Interaction between *M.avium* and HIV drugs is unavoidable in HIV patients as for an improved life expectancy of HIV patients, additional medications have to be administered along with HIV drugs. The anti-*M.avium* drug rifabutin and protease inhibitor, ritonavir are associated with significant drug interactions involving the cytochrome P450 (CYP) enzyme system. Little information is available on the role of various drug delivery strategies in alleviations of interactions between anti-*M.avium* and anti-HIV drugs. The purpose of the present study was to evaluate effect of encapsulation of rifabutin and ritonavir in PLGA nanoparticles on the already known drug interaction exhibited by these drugs.

## Methods

This study was designed including administration of rifabutin and ritonavir singly and in combination in free and nanoencapsulated form to swiss albino mice. Blood samples were taken following drug administration at various time intervals and pharmacokinetic parameters were assessed as: area under plasma drug concentration over time curve (AUC0-∞), mean residence time (MRT) and Cmax etc.

## Results

Overall, nanoencapsulation was observed to avoid the known adverse drug interactions between RBT and RTV in the drug interaction study. (p-value<0.001).

## Conclusion

Our results clearly emphasize the potential of the PLGA nanoparticle formulation to minimize drug interactions as the encapsulated drugs did not result in any significant alteration in kinetic parameters upon co-administration. It can be reasonably stated that nano-encapsulation would not only permit intermittent dosing but also a more favorable pharmacokinetics which further can overcome the drug interactions.

